# Abordagem intersetorial para prevenção e controle da obesidade: a experiência brasileira de 2014 a 2018

**DOI:** 10.26633/RPSP.2019.58

**Published:** 2019-11-26

**Authors:** Mariana Carvalho Pinheiro, Ana Luisa Souza de Paiva Moura, Gisele Ane Bortolini, Janine Giuberti Coutinho, Lilian dos Santos Rahal, Luisete Moraes Bandeira, Michele Lessa de Oliveira, Patrícia Chaves Gentil

**Affiliations:** 1 Ministério da Cidadania Secretaria Especial do Desenvolvimento Social Brasília (DF) Brasil Ministério da Cidadania, Secretaria Especial do Desenvolvimento Social, Brasília (DF), Brasil.; 2 Ministério da Saúde Brasília (DF) Brasil Ministério da Saúde, Brasília (DF), Brasil.; 3 Organização Pan-Americana da Saúde (OPAS) Brasília (DF) Brasil Organização Pan-Americana da Saúde (OPAS), Brasília (DF), Brasil.

**Keywords:** Estratégias, colaboração intersetorial, obesidade, sobrepeso/prevenção e controle, Brasil, Strategies, intersectoral collaboration, obesity, overweight/prevention & control, Brazil, Estrategias, colaboración intersectorial, obesidad, sobrepeso/prevención & control, Brasil

## Abstract

O objetivo do artigo é descrever e comentar as ações implementadas em nível federal, no período de 2014 a 2018, no escopo da Estratégia Intersetorial de Prevenção e Controle da Obesidade, publicada pelo governo federal no Brasil. A Estratégia tem por objetivo prevenir e controlar a obesidade e foi organizada em seis grandes eixos de ação: 1) disponibilidade e acesso a alimentos adequados e saudáveis; 2) educação, comunicação e informação; 3) promoção de modos de vida saudáveis em ambientes específicos/territórios; 4) vigilância alimentar e nutricional, 5) atenção integral à saúde do indivíduo com sobrepeso/obesidade na rede de saúde e 6) regulação e controle da qualidade e inocuidade de alimentos. Para levantamento das informações, questionários semiestruturados foram enviados a representantes dos Ministérios da Educação, Saúde, Planejamento, Cidadania e Desenvolvimento Agrário. As ações implementadas são apresentadas segundo os eixos da Estratégia. Resultados positivos foram alcançados a partir do modelo de coordenação adotado, com gestão compartilhada, ainda que desafios necessitem ser superados. A agenda de ações propostas na Estratégia está alinhada ao contexto brasileiro e o modelo intersetorial de gestão permanece vigente até o período de elaboração do artigo.

## Abstract (en)

The present article aims to describe and discuss the actions implemented from 2014 to 2018 at the federal level in the context of the Intersectoral Strategy to Prevent and Control Obesity issued by the Brazilian federal government. The goal of the Intersectoral Strategy is to prevent and control obesity based on six action plans: 1) availability and access to adequate and healthy foods; 2) education, communication, and information; 3) promotion of healthy lifestyles in specific environments/territories; 4) food and nutrition surveillance; 5) comprehensive care for individuals presenting obesity in the health care network; and 6) regulation and control of the quality and harmlessness of food. To gather information, semi-structured questionnaires were submitted to representatives from the ministries of Education, Health, Planning, Citizenship, and Agrarian Development. The actions implemented in the period were described in terms of the six action plans. Positive results were achieved through the shared management model adopted, even though many challenges still remain. The agenda for action proposed in the Intersectoral Strategy is attuned to the Brazilian context, and intersectoral management was still in place until the writing of the present article.

## RESUMEN

El objetivo del presente artículo es describir las actividades realizadas a nivel federal en el período 2014-2018 dentro del marco de la estrategia intersectorial de prevención y control de la Obesidad, publicada por el Gobierno Federal de Brasil, y hacer algunos comentarios pertinentes. La estrategia tiene por objetivo prevenir y controlar la obesidad y se ha organizado en seis grandes ejes de acción: 1) disponibilidad de alimentos adecuados y saludables y acceso a los mismos; 2) educación, comunicación e información; 3) promoción de estilos de vida saludables en entornos y territorios específicos; 4) vigilancia alimentaria y nutricional; 5) atención integral a la salud de las personas con sobrepeso y obesidad en la red asistencial, y 6) regulación y control de la calidad y la inocuidad de los alimentos. Para recopilar información se enviaron cuestionarios semiestructurados a representantes de los Ministerios de Educación, Salud, Planificación, Ciudadanía y Desarrollo Agrario. Las actividades realizadas se presentan según los ejes de acción de la estrategia. Se lograron resultados positivos a partir del modelo de coordinación adoptado, con gestión compartida, aunque fue preciso superar varios desafíos. El programa de trabajo sobre los ejes de acción propuestos en la estrategia está en consonancia con el contexto brasileño y el modelo intersectorial de gestión permaneció vigente hasta el momento de preparación de este artículo.

O excesso de peso apresenta tendência de aumento no Brasil. Em homens, a prevalência do excesso de peso cresceu de 47,5% em 2006 para 57,3% em 2017. Em mulheres, a prevalência cresceu de 38,5% para 51,2% (1) no mesmo período. Em crianças de 5 a 9 anos, o excesso de peso atinge 33,5% (2) e, em adolescentes, 17,1% (3). Esses dados acompanham a tendência da América Latina e Caribe, onde cerca de 58% da população adulta apresentam excesso de peso (4).

As causas do excesso de peso são multifatoriais e complexas. No Brasil, o desenho das políticas públicas reconhece que os ambientes exercem um papel preponderante nessa determinação. Nessa lógica, a ação governamental é essencial para promover ambientes alimentares saudáveis, reduzir a obesidade e garantir a segurança alimentar e nutricional da população (5). Essa perspectiva tem sido adotada no país desde a criação do Sistema Nacional de Segurança Alimentar e Nutricional (SISAN), que reconhece a intersetorialidade como elemento fundamental para promover o Direito Humano à Alimentação Adequada (6). O SISAN engloba uma Câmara Interministerial de Segurança Alimentar e Nutricional (CAISAN), responsável pela articulação de 20 ministérios que realizam a gestão, financiamento e execução do Plano Nacional de Segurança Alimentar e Nutricional (7).

A problemática da relação entre excesso de peso e doenças crônicas não transmissíveis (DCNT) resultou no desenvolvimento de diversas agendas intersetoriais, como o Plano de Ações Estratégicas para o Enfrentamento das DCNT (8) e I e II Planos Nacionais de Segurança Alimentar e Nutricional (9, 10). Além disso, estabeleceu-se a Estratégia Intersetorial de Prevenção e Controle da Obesidade (11), gerida de forma intersetorial por meio de um comitê técnico na CAISAN (12).

A Estratégia Intersetorial contempla um conjunto de ações governamentais para prevenir e controlar a obesidade na população brasileira e foi organizada em seis grandes eixos de ação: 1) disponibilidade e acesso a alimentos adequados e saudáveis; 2) educação, comunicação e informação; 3) promoção de modos de vida saudáveis em ambientes específicos/territórios; 4) vigilância alimentar e nutricional; 5) atenção integral à saúde do indivíduo com sobrepeso/obesidade na rede de saúde; e 6) regulação e controle da qualidade e inocuidade de alimentos (11).

Internacionalmente, o Brasil firmou acordo para o enfrentamento de todas as formas da má nutrição no âmbito do Mercosul e no contexto da Década de Ação das Nações Unidas para a Nutrição (2016-2025) (13). Como o SISAN, a Década reconhece que as principais causas da má nutrição são complexas e multifatoriais, exigindo coordenação intersetorial e o envolvimento de diversos atores por meio de sistemas de governança transversais e coerentes (14). Assim, os planos e prioridades em segurança alimentar e nutricional no Brasil reafirmam o compromisso com o enfrentamento do excesso de peso e da obesidade: deter o crescimento da obesidade na população adulta, reduzir em pelo menos 30% o consumo regular de refrigerante e suco artificial e ampliar em no mínimo 17,8% o percentual de adultos que consomem frutas e hortaliças regularmente (9, 13).

Diante da necessidade de coordenar e impulsionar esforços intersetoriais, este artigo apresenta uma análise das ações implementadas no período de 2014 a 2018 pelos setores envolvidos no âmbito da Estratégia Intersetorial de Prevenção e Controle da Obesidade.

## LEVANTAMENTO DAS INFORMAÇÕES

Realizou-se um levantamento transversal das ações de prevenção e controle da obesidade implementadas por diferentes órgãos do governo brasileiro no período de 2014 a 2018. Foram enviados questionários semiestrurados, em formato eletrônico, aos coordenadores das áreas técnicas responsáveis pelas ações da Estratégia Intersetorial de Prevenção e Controle da Obesidade nos Ministérios da Educação, Saúde, Planejamento, Cidadania e Desenvolvimento Agrário, os quais integram o Comitê Técnico. O instrumento foi organizado e testado de acordo com os eixos da Estratégia e a atuação de cada órgão. O objetivo foi acompanhar as ações de prevenção e controle da obesidade realizadas por esses setores no período. Foram respondidas perguntas sobre o planejamento estratégico de cada órgão, incluindo objetivos e metas relacionadas à prevenção e ao controle da obesidade; parcerias estabelecidas; participação social nessas ações; e avaliação do Comitê Técnico.

O instrumento foi enviado em setembro de 2017 para levantamento das ações no biênio 2014-2016 e em agosto de 2018 para atualização. As respostas foram sistematizadas de acordo com os eixos da Estratégia.

## EIXO 1: DISPONIBILIDADE E ACESSO A ALIMENTOS ADEQUADOS E SAUDÁVEIS

Este eixo reúne ações que oportunizam o acesso físico a alimentos e preparações tradicionais. Engloba ações de produção de alimentos da agricultura familiar, oferta e preparações nos diferentes espaços institucionais e nos equipamentos públicos. Ao considerar este eixo, é importante salientar que o Brasil dispõe de programas para facilitar o acesso a alimentos pelas famílias e populações vulneráveis, seja pela transferência de renda, seja pela disponibilização de alimentos. Por exemplo, o Programa Bolsa Família (http://mds.gov.br/assuntos/bolsa-familia) beneficia 14 milhões de famílias vulneráveis; e o Benefício de Prestação Continuada (http://mds.gov.br/assuntos/assistencia-social/beneficios-assistenciais/bpc) é um direito constitucional que assegura salário mínimo mensal a idosos e pessoas com deficiência.

Para atender o eixo 1, equipamentos públicos de segurança alimentar e nutricional, como restaurantes populares e bancos de alimentos, foram implantados, modernizados e equipados. As unidades de apoio à distribuição de alimentos da agricultura familiar receberam veículos e foram equipadas para apoiar a distribuição de alimentos em escolas, entidades socioassistenciais e mercados locais e regionais. Em 2017, foi publicada a Estratégia Intersetorial para a Redução de Perdas e Desperdício de Alimentos no Brasil, no contexto da segurança alimentar e nutricional e da garantia do Direito Humano à Alimentação Adequada, fortalecendo a integração dos bancos de alimentos no País (15).

No campo da oferta, o Programa de Aquisição de Alimentos adquire alimentos produzidos pela agricultura familiar com dispensa de licitação e os destina às pessoas em situação de insegurança alimentar e nutricional assistidas pelas redes socioassistencial e de ensino. As compras públicas de produtos da agricultura familiar ultrapassaram o valor de 1,5 bilhão de dólares de 2014 a 2016. Cerca de 60% desse valor foram destinados à alimentação escolar. Existe obrigatoriedade, desde 2015, de os órgãos federais destinarem 30% dos recursos para compras da agricultura familiar, modelo implementado pelas forças armadas e por algumas universidades (16).

O Programa Nacional de Alimentação Escolar (PNAE) oferece diariamente refeições para mais de 40 milhões de escolares (2016/2017) da rede de educação básica. Em 2016, 23,9% do total do recurso foram usados para aquisição de alimentos da agricultura familiar; a meta é alcançar, em 2019, o percentual de 30% fixado em lei. Em 2017 e 2018, foram revisados os parâmetros nutricionais do PNAE para alinhamento ao Guia Alimentar para a População Brasileira (17).

O Pacto Nacional pela Alimentação Saudável foi instituído em 2015, incentivando estados e municípios na proposição de ações locais de ampliação da oferta, disponibilidade e consumo de alimentos saudáveis e combate ao excesso de peso (18). Cinco unidades da federação aderiram (Distrito Federal, Alagoas, Maranhão, Paraíba e Sergipe). Em 2018, no contexto da Década de Ação para a Nutrição, o Brasil assumiu o papel de coordenar redes de ação com os temas governança em segurança alimentar e nutricional e compras públicas da agricultura familiar. As redes de ação são espaços de intercâmbio e colaboração entre países das Américas (13).

As ações apresentadas evidenciam o esforço intersetorial para estabelecer sistemas alimentares saudáveis e sustentáveis, com avanços importantes nas políticas de apoio à agricultura familiar, sobretudo a política de compras públicas. As compras públicas permitem a oferta de alimentos frescos, variados e nutritivos, melhorando a qualidade da alimentação para a população. Além disso, possibilitam a abertura de novos mercados para a agricultura familiar, estabelecimento de marcos legais mais justos aos agricultores, participação social, desenvolvimento local, articulação de políticas públicas e valorização da agricultura familiar na sociedade (19).

## EIXO 2: AÇÕES DE EDUCAÇÃO, COMUNICAÇÃO E INFORMAÇÃO

O eixo 2 envolve ações de compartilhamento de conhecimentos e práticas que contribuam para a melhoria das condições de vida, saúde e segurança alimentar e nutricional da população – desde o acesso a informações e estímulo ao autocuidado até estratégias articuladas e contínuas de educação e de mobilização da opinião pública. Em 2014, foi publicado o Guia Alimentar da População Brasileira pelo Ministério da Saúde (17), referência oficial de recomendações sobre o tema no país. Com uma abordagem baseada no nível de processamento dos alimentos, o Guia é uma ferramenta para melhorar os padrões de consumo e bem-estar nutricional individual e das populações (20). Foram produzidos diferentes materiais de apoio à divulgação e implementação do Guia. O Guia Alimentar para Crianças Menores de 2 Anos, publicado em sua primeira versão em 2002, está em atualização. O Ministério da Saúde também assumiu a coordenação da Rede de Guias no contexto da Década, para incentivar a elaboração, implementação, monitoramento e avaliação de guias alimentares baseados no nível de processamento de alimentos por países das Américas.

No período, o Ministério da Cidadania realizou formação em educação alimentar e nutricional e compras públicas da agricultura para profissionais, gestores e agricultores em todo o País. A Secretaria Especial de Agricultura Familiar e do Desenvolvimento Agrário (SEAD) também publicou materiais sobre segurança alimentar e nutricional para agentes técnicos rurais e ampliou a participação da agricultura familiar na rede *Slow Food* (http://www.slowfoodbrasil.com/), formada por profissionais de diversas áreas que almejam transformar o território com a valorização do alimento.

As ações do eixo 2 demonstram o estabelecimento de marcos referenciais que apoiam a agenda da alimentação saudável no país. Essa construção é fruto do diálogo entre diferentes setores e grupos sociais. Envolve estratégias de divulgação e processos educativos que buscam harmonizar o saber técnico, popular e cultural para ampliar a compreensão e fomentar ambientes alimentares saudáveis e sustentáveis, assim como capacidades individuais para a adoção de práticas alimentares saudáveis.

## EIXO 3: PROMOÇÃO DE MODOS DE VIDA SAUDÁVEIS NOS AMBIENTES/TERRITÓRIOS

O eixo 3 fomenta o estabelecimento de espaços para ações educativas e de promoção à saúde para promover e facilitar práticas alimentares saudáveis, com destaque aos espaços urbanos e institucionais (ambientes de trabalho, escolar, redes de saúde e socioassistencial).

Para promover o aleitamento materno e a alimentação complementar saudável, o Ministério da Saúde coordena a Estratégia Amamenta e Alimenta Brasil, com qualificação da atuação de mais de 42 mil profissionais de saúde da atenção básica e formação de mais de 5 mil tutores no período. O Brasil conta ainda com Rede de Bancos de Leite Humano e mais de 380 Hospitais Amigos da Criança. Essas ações respaldam as recomendações do Plano de Ação para Prevenção da Obesidade em crianças e adolescentes (4).

No desenvolvimento de ações e estratégias de promoção da saúde e do cuidado, investiu-se na implantação de polos de Academia da Saúde, financiados pelo governo federal, com infraestrutura, equipamentos e profissionais qualificados e conectados aos demais pontos da rede de saúde. Estão em funcionamento 1 443 polos, com mais 2 468 concluídos ou em construção em todo o país. Promover e facilitar a realização de atividades físicas é uma das principais políticas para transformação do ambiente obesogênico (20).

O tema da alimentação saudável e promoção de práticas corporais foi incluído no Programa Saúde na Escola (PSE), uma agenda do Ministério da Saúde e do Ministério da Educação que desde 2007 buscam contribuir para a formação integral dos estudantes da rede pública de educação básica por meio de ações de prevenção, promoção e atenção à saúde. Atualmente, o PSE está em 5 040 municípios (90,5% do total), com envolvimento de 36 mil equipes de saúde e mais de 20 milhões de alunos em 85 mil escolas.

No âmbito do PNAE, foram ofertadas capacitações e divulgação de materiais de incentivo à redução de alimentos ultraprocessados na alimentação escolar. Foram também inseridos conteúdos de alimentação saudável na contracapa dos livros didáticos para as escolas públicas do país, com distribuição gratuita de mais de 89 milhões de livros. No campo da assistência social, em torno de 2 mil profissionais foram formados na agenda de alimentação saudável.

Para o ambiente de trabalho, o Ministério do Planejamento publicou, em 2016, normativa para estimular gestores públicos federais a promoverem ações de educação alimentar e nutricional em todos os órgãos e entidades da administração pública (21). No mesmo ano, o Ministério da Saúde publicou portaria que restringiu a venda e a oferta de alimentos ultraprocessados nas dependências do órgão (22).

Atividades de educação alimentar e nutricional nos ambientes institucionais precisam ser complementadas por ações para melhoria da qualidade da oferta de alimentos, apoiando práticas saudáveis. Importantes avanços foram obtidos no período, especialmente nos campos escolar e da saúde. Dada a dimensão territorial brasileira, os ambientes de trabalho e a rede socioassistencial dispõem de grande potencial de expansão.

## EIXO 4: VIGILÂNCIA ALIMENTAR E NUTRICIONAL

A avaliação contínua do perfil alimentar e nutricional da população e seus fatores determinantes compõem a vigilância alimentar e nutricional. No Brasil, recomenda-se que os serviços de saúde de atenção básica realizem avaliação de consumo alimentar e antropometria de indivíduos de todas as fases da vida por meio do Sistema de Vigilância Alimentar e Nutricional (SisvanWeb), de forma integrada com informações de outras pesquisas, inquéritos e sistemas.

Registrou-se no SisvanWeb, em 2017, cobertura de 14,1% da população pelas equipes de saúde, isto é: mais de 27 milhões de indivíduos tiveram seu estado nutricional acompanhado. O Ministério da Saúde financiou a aquisição de equipamentos antropométricos e disponibilizou cursos *on-line*. Realiza também inquéritos anuais de abrangência nacional para vigilância de fatores de risco e proteção para doenças crônicas por inquérito telefônico; Pesquisa Nacional de Saúde Escolar; Pesquisa de Orçamentos Familiares; Pesquisa Nacional de Saúde; e Pesquisa Nacional de Demografia e Saúde da Criança e da Mulher. Está em curso o I Inquérito de Alimentação e Nutrição Infantil, em âmbito nacional, que contempla medidas antropométricas, consumo alimentar e análise bioquímica de menores de 5 anos.

A vigilância é fundamental para orientar a formulação de políticas locais, regionais e nacionais, permitindo ainda sensibilidade a questões de equidade (4). A efetivação da vigilância alimentar e nutricional, local e nacionalmente, requer a combinação de estratégias que permitam reconhecer as condições de saúde e nutrição da população, bem como das morbidades associadas.

## EIXO 5: ATENÇÃO INTEGRAL À SAÚDE DO INDIVÍDUO COM SOBREPESO/ OBESIDADE NA REDE DE SAÚDE

Em 2017 foi lançado o Programa Crescer Saudável, conjunto de ações articuladas entre saúde e educação para promoção e proteção da saúde, diagnóstico e tratamento da obesidade, incentivo à prática corporal e de atividade física e ações voltadas à mudança de práticas não saudáveis. Para participar do Programa, foram priorizados municípios com maior porte populacional, maior cobertura de vigilância alimentar e nutricional e maior prevalência de excesso de peso em menores de 10 anos.

O Ministério da Saúde apoiou ainda os estados na organização do cuidado integral aos indivíduos com excesso de peso. Foram instituídas diretrizes de incentivo à organização local de linhas de cuidado de sobrepeso e obesidade, com definição de fluxos para assistir o usuário e organizar os serviços e ações a serem desenvolvidas nos pontos de atenção da rede, segundo níveis de complexidade. Dos 27 estados brasileiros, 20 estão em fase de implantação dessas linhas.

Para qualificação do cuidado ofertado pelos profissionais de saúde, o Ministério da Saúde está finalizando cursos de aperfeiçoamento no manejo da obesidade e elaborou protocolos clínicos de tratamento. Projetos de pesquisa, extensão e formação de profissionais de 22 instituições de ensino foram também selecionados.

Ações de diagnóstico, prevenção e tratamento da obesidade requerem a integração e a organização dos serviços de diferentes complexidades, em articulação com outros equipamentos do território, contemplando indivíduos, famílias e comunidades e considerando as diferentes fases da vida, questões de gênero e especificidades de grupos e povos tradicionais. O modelo do sistema de saúde brasileiro, universal, público e integral, tem, portanto, um conjunto de desafios para a adequada atenção à saúde dos usuários com sobrepeso e obesidade. Investir na qualificação dos profissionais da atenção básica de saúde quanto á prevenção e ao controle dos agravos nutricionais foi uma das prioridades elencadas para revisão do Plano Nacional de Segurança Alimentar e Nutricional 2016-2019 (21).

## EIXO 6: REGULAÇÃO E CONTROLE DA QUALIDADE E INOCUIDADE DE ALIMENTOS

Este eixo trata de agendas regulatórias da rotulagem nutricional, venda de alimentos e bebidas nos espaços escolares, publicidade de alimentos e taxação de bebidas açucaradas. A CAISAN participou ativamente do processo de discussão, junto à Agência Nacional de Vigilância Sanitária (ANVISA) – órgão responsável pela normatização e regulamentação de alimentos –, do modelo de rotulagem de advertência para alimentos com quantidades elevadas de calorias, açúcares, gorduras, sódio, edulcorantes e aditivos alimentares, baseado no Modelo de Perfil de Nutrientes da Organização Pan-Americana da Saúde (OPAS). O relatório de análise de impacto regulatório da ANVISA concluiu que modelos de advertência se mostraram mais efetivos segundo evidências técnico-científicas disponíveis (23, 24). No momento, aguarda-se a publicação de consulta pública pela Agência com a proposta do modelo brasileiro.

Acerca da regulamentação da venda de alimentos e bebidas em cantinas escolares, foi elaborado projeto de lei que regulamenta a comercialização e publicidade de alimentos ultraprocessados com altos teores de açúcar, gordura e sódio nesses espaços. No campo da regulamentação da publicidade de alimentos e bebidas, o Ministério da Saúde propôs um projeto de lei que solicita a inclusão de mensagens educativas sobre nutrientes críticos de produtos ultraprocessados na publicidade de alimentos para adultos, além de proibir a publicidade para crianças menores de 12 anos. Sobre ambos os temas, há projetos de lei tramitando no Congresso Nacional, de autoria de diferentes deputados e senadores. São temas que requerem grande incidência e *advocacy* no âmbito do parlamento brasileiro.

Sobre a taxação de bebidas açucaradas, desde 2015 foram realizados eventos e reuniões técnicas com diferentes representações (Ministério da Fazenda, Receita Federal, universidades e entidades da sociedade civil) para proposição de medidas fiscais como estratégia de desestímulo ao consumo desses produtos. Em 2018 foram publicados decretos que reduziram o crédito tributário recebido pelas empresas produtoras de concentrados de refrigerantes no Brasil, com redução das distorções tributárias existentes e consequente aumento de preço. O Brasil tem grandes distorções fiscais que precisam ser resolvidas. Novos estudos estão em andamento, assim como tramitam projetos de lei com posições antagônicas sobre a questão. Este tema ainda é um grande desafio e a agenda econômica precisa considerar os efeitos da política fiscal para a saúde pública do país.

O Plano Nacional de Redução do Sódio em Alimentos Processados, como parte da agenda de redução de risco e reformulação da composição de alimentos, inclui acordos voluntários entre o Ministério da Saúde e associações do setor de alimentos. Determina redução gradual de sódio nas diferentes categorias de alimentos selecionadas com base na sua contribuição na dieta dos brasileiros. O monitoramento das metas mostrou que, de 1 962 produtos avaliados, 1 771 cumpriram as metas pactuadas (90,3%). Os resultados são positivos e fortalecedores, fazendo do Brasil referência para esta agenda nas Américas. Na agenda de redução dos teores de açúcar em alimentos processados e ultraprocessados, foram definidas as categorias de gêneros prioritários, segundo sua contribuição na dieta da população brasileira, além da discussão do banimento do uso, no País, de gordura trans em alimentos industrializados.

As ações regulatórias buscam definir limites entre sociedade e Estado e entre governo e mercado na garantia dos direitos constitucionais e do bem público. Assim, criam diretrizes para a atuação dos setores comerciais e objetivam proteger a população contra práticas abusivas, especialmente oriundas do setor privado comercial (19). Agências internacionais, governos, consumidores, empresas e instituições científicas desempenham papéis estratégicos e participam da definição de alternativas por meio de diferentes mecanismos e arenas. Desequilíbrios de poder e informação, complexidade das normativas, multiplicidade de atores envolvidos e distintos mecanismos de pressão exercidos por corporações com alto poder de influência no cenário econômico frequentemente exercem forte impacto no processo decisório, tornando o ambiente conflituoso e a agenda desafiadora. Regular a produção ou desincentivar o consumo de ultraprocessados é uma das principais políticas recomendadas pela OPAS e OMS para melhoria do ambiente alimentar (4). As ações regulatórias foram fortemente pautadas como prioritárias para a revisão do Plano Nacional de Segurança Alimentar e Nutricional 2016-2019 (10, 25). A tendência de enfraquecimento do papel dos governos na formulação e implementação de medidas restritivas é reconhecida pela OMS desde 2004 e, desde então, no Brasil, essas iniciativas são retomadas de tempo em tempo, mas com pouca efetividade.

## CONCLUSÕES

A implementação da Estratégia brasileira tem como pressuposto a intersetorialidade das ações de prevenção, enfrentamento e controle da obesidade. A coordenação intersetorial dessas ações requer a instituição de mecanismos de governança que permitam o diálogo entre diversos setores, conjuguem esforços e evitem a sobreposição de ações. Formular e implementar políticas públicas com essa abordagem é uma recomendação dos organismos internacionais para combater a epidemia de obesidade (20).

Compreendendo a problemática multicausal da obesidade, espaços de coordenação entre os setores devem ser promovidos para permitir o alinhamento institucional e combinar ações (4). A percepção do sistema alimentar como determinante do excesso de peso e a maior efetividade do conjunto de ações de prevenção e controle da obesidade infantil exigem estratégias intersetoriais (19). Nesse sentido, a convergência de objetivos da Estratégia é percebida e também defendida por Henriques et al. (19) com a criação do espaço político de gestão compartilhada e participação social na política de segurança alimentar e nutricional no âmbito da CAISAN.

A integração das políticas de segurança alimentar e nutricional e, mais especificamente, de prevenção de controle da obesidade, associada à criação de mecanismos de monitoramento, é prerrogativa defendida também pelas representações de controle social no país (21). Monitoramento, avaliação, comunicação, informação, mobilização e *advocacy* da Estratégia são etapas que permitem considerar o modelo de gestão governamental adotado como exitoso e eficaz.

Importantes avanços com o modelo adotado pela CAISAN desde 2014 foram percebidos, ainda que alguns eixos apresentem grandes desafios a serem superados no estabelecimento de ambientes mais favoráveis à adoção de práticas alimentares saudáveis, como os condicionantes de produção, comercialização e acesso a alimentos e as questões regulatórias. A efetiva implementação de tais medidas demanda graus distintos de recursos políticos, pois afeta, também de forma distinta, os interesses em disputa (19). De outro lado, temáticas aprofundaram-se, como a discussão sobre perdas e desperdícios de alimentos. Ainda assim, a agenda de ações permanece alinhada ao contexto brasileiro e permitiu à gestão coordenadora manter-se atualizada ao longo do período estudado.

## Contribuição dos autores.

MCP, ALSPM, GAB, JGC, LSR, LMB, MLO e PCG elaboraram o projeto, redigiram a primeira versão do artigo, realizaram a coleta de dados, analisaram os dados, interpretaram os resultados e redigiram a versão final do artigo. Todas as autoras revisaram e aprovaram a versão definitiva.

## Agradecimentos.

À consultora Natália Tenuta pelo apoio no planejamento, coleta e análise preliminar dos dados e aos representantes do Comitê Técnico de Prevenção e Controle da Obesidade.

## Declaração.

As opiniões expressas no manuscrito são de responsabilidade exclusiva dos autores e não refletem necessariamente a opinião ou política da RPSP/PAJPH ou da Organização Pan-Americana da Saúde (OPAS).
